# Surface chemistry on a polarizable surface: Coupling of CO with KTaO_3_(001)

**DOI:** 10.1126/sciadv.abq1433

**Published:** 2022-08-19

**Authors:** Zhichang Wang, Michele Reticcioli, Zdenek Jakub, Igor Sokolović, Matthias Meier, Lynn A. Boatner, Michael Schmid, Gareth S. Parkinson, Ulrike Diebold, Cesare Franchini, Martin Setvin

**Affiliations:** ^1^ Institute of Applied Physics, TU Wien, Vienna, Austria.; ^2^ State Key Laboratory for Physical Chemistry of Solid Surfaces, Collaborative Innovation Center of Chemistry for Energy Materials, and Department of Chemistry, College of Chemistry and Chemical Engineering, Xiamen University, Xiamen, China.; ^3^ Faculty of Physics and Center for Computational Materials Science, University of Vienna, Vienna, Austria.; ^4^ Materials Science and Technology Division, Oak Ridge National Laboratory, Oak Ridge, TN 37831, USA.; ^5^ Dipartimento di Fisica e Astronomia, Universita di Bologna, 40127 Bologna, Italy.; ^6^ Department of Surface and Plasma Science, Faculty of Mathematics and Physics, Charles University, 180 00 Prague 8, Czech Republic.

## Abstract

Polarizable materials attract attention in catalysis because they have a free parameter for tuning chemical reactivity. Their surfaces entangle the dielectric polarization with surface polarity, excess charge, and orbital hybridization. How this affects individual adsorbed molecules is shown for the incipient ferroelectric perovskite KTaO_3_. This intrinsically polar material cleaves along (001) into KO- and TaO_2_-terminated surface domains. At TaO_2_ terraces, the polarity-compensating excess electrons form a two-dimensional electron gas and can also localize by coupling to ferroelectric distortions. TaO_2_ terraces host two distinct types of CO molecules, adsorbed at equivalent lattice sites but charged differently as seen in atomic force microscopy/scanning tunneling microscopy. Temperature-programmed desorption shows substantially stronger binding of the charged CO; in density functional theory calculations, the excess charge favors a bipolaronic configuration coupled to the CO. These results pinpoint how adsorption states couple to ferroelectric polarization.

## Teaser

Coupling of carbon monoxide with ferroelectric lattice distortions of KTaO_3_(001) chemically activates the molecule.

## INTRODUCTION

Ferroelectricity is the ability of materials to spontaneously adopt electric polarization. It is often present in perovskites (general formula ABO_3_) (*1*), where the polarization predominantly stems from the displacement of the octahedrally coordinated B atoms (*2*). B is typically a transition metal, and its ferroelectric displacement is associated with changes in bonding angles and orbital hybridization, hence electronic and chemical properties (*3*). Ferroelectric polarization is a promising tool in catalysis (*4*–*11*), offering the option of spatial charge separation of photochemical reactions (*12*–*14*), tuning the bonding strength of adsorbates, and shifting scaling relations. The transition between the paraelectric and ferroelectric phases in a crystal’s bulk renders its surfaces polar (*15*, *16*), a phenomenon that is increasingly used for powering redox reactions in fuel generation or self-cleaning applications (*10*, *17*): Polar surfaces can promote redox reactions by providing excess electrons or holes by means of the polar catastrophe (*18*). Cycling the temperature through *T*_C_ (ferroelectric Curie temperature), poling direction, or applying periodic mechanical strain (i.e., via ultrasonic irradiation) allows running catalytic reactions continuously. In an appealing strategy, it was proposed (*19*) that the Sabatier principle for efficient catalysis (i.e., the compromise between strong adsorption to bind and activate the reactants and weak adsorption to allow desorption of the products) could be overcome by toggling the electric polarization, thus switching between weak and strong adsorption.

Testing the fundamental properties of materials is best done with well-defined samples. Cleaved KTaO_3_(001) (*20*, *21*) was used in this study because its bulk-terminated structure is consistent with the widely accepted picture of surfaces prepared by wet chemical techniques (*22*). Moreover, KTaO_3_(001) has uncompensated polarity (*20*), which produces surplus electrons at its TaO_2_ terraces. KTaO_3_ is an incipient ferroelectric; it does not spontaneously polarize in the bulk, but the Ta atoms can substantially move from their equilibrium positions with little energy cost (*2*).

In this work, we use carbon monoxide to probe whether and how ferroelectric lattice distortions couple to adsorbates. Combined atomic force microscopy/scanning tunneling microscopy (AFM/STM) is used to identify the adsorption configurations of CO, temperature-programmed desorption (TPD) determines the desorption kinetics and energies, and density functional theory (DFT) calculations provide insights into the coupling with ferroelectric lattice distortions. This altogether identifies the key processes that dictate the surface chemistry on polarizable materials.

## RESULTS

**Fig. 1.**
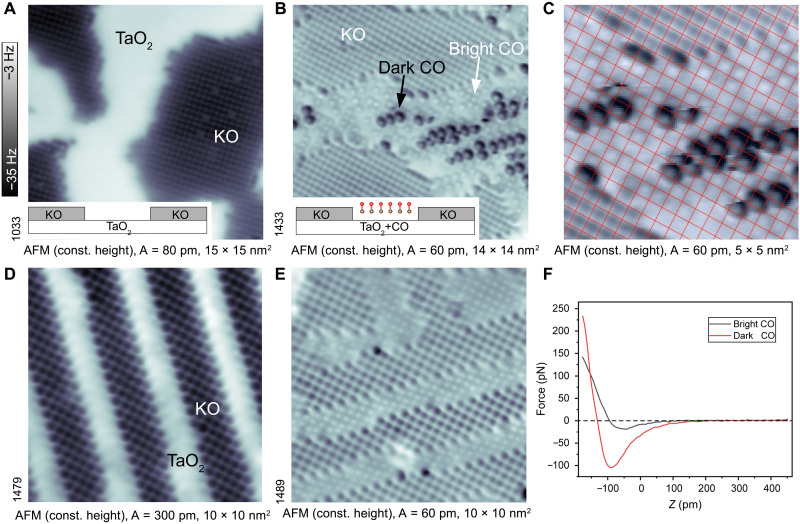
CO adsorption on bulk-terminated KTaO_3_(001). (**A** and **B**) Atomically resolved, constant-height AFM images (*T* = 4.8 K) of the as-cleaved surface before and after exposure to CO (dosed 1 Langmuir at 92 K), which results in CO adsorbed at the TaO_2_ termination. Note the two CO types: dark and bright. The insets show a schematic side view of the terrace arrangement. (**C**) Zoom-in AFM image with square lattice superimposed; both CO species sit at Ta sites. (**D** and **E**) AFM images of the surface, restructured to smaller, aligned terraces by annealing to 470 K (*20*) before and after CO adsorption, respectively. (**F**) Force-distance curves showing the short-range forces measured above the bright and dark CO. All images were measured at sample voltage (*V*_S_) = 0 V.

A typical as-cleaved KTaO_3_(001) surface is shown in the constant-height noncontact AFM image in Fig. 1A. The surface consists of domains of KO and TaO_2_ terminations, where the KO always lies one-half unit cell higher with respect to the TaO_2_ (*20*). The lower-lying TaO_2_ termination appears unresolved in the AFM images due to a weaker attractive force, while the regions with atomic resolution correspond to KO. Previous works (*20*) have shown that larger TaO_2_ terraces (>2 nm size) host a high density of electrons that arise from uncompensated polarity (*18*), producing a two-dimensional (2D) electron gas (*23*), charge density waves, polarons, and bipolarons (Ta atoms with one or two excess electrons, changing the occupation from 5d^0^ to 5d^1^ or to 5d^2^, respectively) (*24*, *25*). The as-cleaved KTaO_3_(001) surface is unstable, and annealing leads to restructuring the KO/TaO_2_ terraces into a labyrinth of ~2-nm-wide narrow stripes (see Fig. 1, D and E) and absence of in-gap states.

Figure 1B shows the surface after dosing 1 Langmuir (1 L = 1.33 × 10^−4^ Pa·s) of CO at *T* = 92 K. At this temperature, CO adsorbs only on the TaO_2_ termination, at well-defined lattice sites. Adsorption on KO is weaker and incommensurate with the substrate (see fig. S1). The following focuses on adsorption on the TaO_2_ terraces only, because calculations attribute the catalytic activity of perovskites predominantly to the transition metal cations (*26*).

The AFM image in Fig. 1B shows two distinctly different CO species referred to as “dark” and “bright.” The contrast in AFM is chemical in nature, i.e., it reflects the interaction between the CO-terminated tip and the adsorbed CO (see Fig. 1F). While the bright CO interacts only weakly with the tip, the dark CO shows a considerable attractive force before entering the repulsive regime (distance-dependent images are shown in fig. S2). Both types of CO species are located at equivalent lattice positions, i.e., above surface Ta atoms (see the grid overlaid image in Fig. 1C). The dark CO molecules only appear at terraces that exceed a critical size of about 2 nm. This is illustrated in Fig. 1E, where the TaO_2_ terraces are 1 to 2 nm wide. Here, the surface was first annealed to 470 K to restructure the two terminations into thinner stripes (*20*), and CO was dosed subsequently. All the CO molecules appear bright, and the dark CO is absent on these small TaO_2_ terraces.

**Fig. 2.**
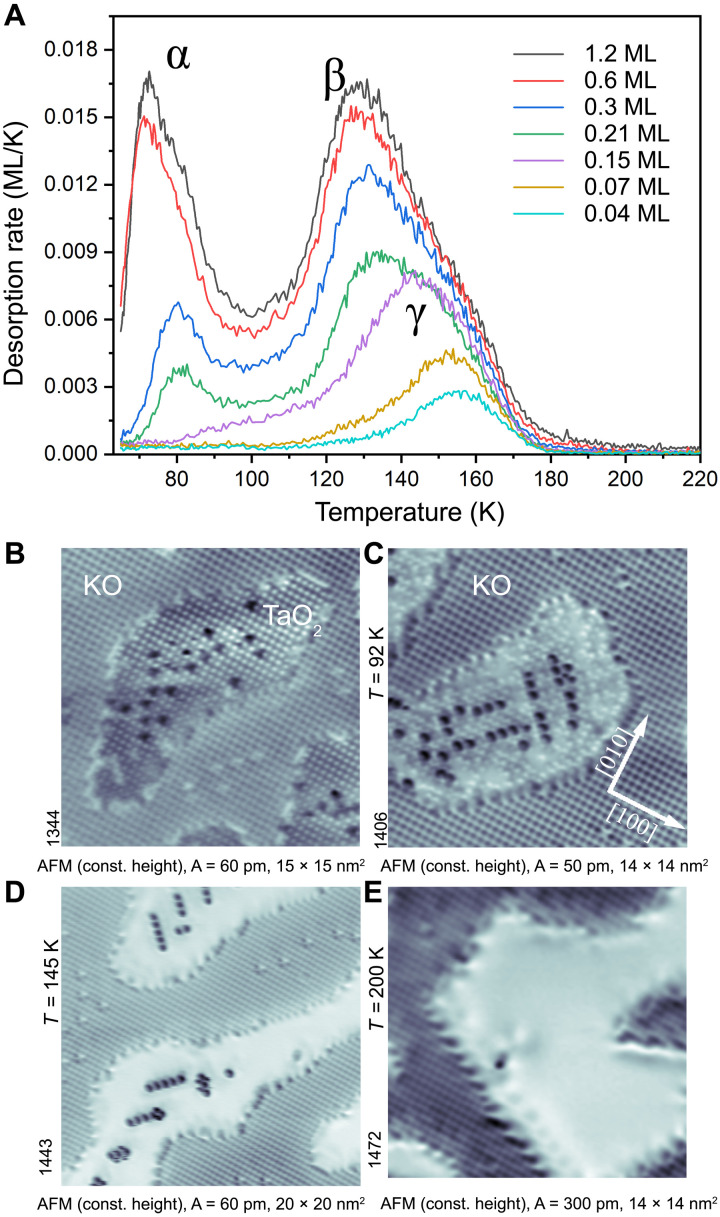
Desorption behavior. (**A**) TPD spectra of CO adsorbed on as-cleaved KTaO_3_(001) for various exposures, given in monolayers (ML). The three desorption peaks labeled α, β, and γ refer to CO adsorbed on KO and the bright and dark CO on TaO_2_, respectively. This is apparent from the AFM images in (**B** to **E**), taken at 4.8 K, after annealing the CO-covered surface to 85, 92, 145, and 200 K, respectively. Note that the CO only adsorbs on the TaO_2_ termination in all images. The KO terraces are clean; the bright dots are attributed to oxygen vacancies (*20*).

TPD was used to quantify the desorption energy of the CO species (data measured on the as-cleaved surfaces; see Fig. 2). Desorption traces obtained with ^13^CO doses ranging from 0.07 to 2 L are shown in Fig. 2A. The three desorption peaks centered at ~75, ~130, and ~155 K are labeled as α, β, and γ, respectively. On the basis of AFM images obtained after annealing to specific temperatures, these desorption peaks are assigned to different CO configurations (see Fig. 2, B to E). The α-peak originates from the KO terraces (see fig. S1), while β and γ originate from TaO_2_. When dosing at 85 K, CO adsorbs only on the TaO_2_, with a densely packed layer in two different adsorption configurations (Fig. 2B). Annealing to 92 K resulted in desorption of some of the bright CO molecules (Fig. 2C). The dark CO tends to align along the <110> directions, with a spacing between the rows of approximately 1.6 nm (Fig. 2C). A different example of this ordering is shown in fig. S3. After annealing to 145 K, all bright CO has desorbed and only dark CO remained at the TaO_2_ (Fig. 2D). Annealing to 200 K resulted in desorption of all the remaining CO. The β and γ desorption peaks therefore correspond to the bright and dark CO, respectively.

For CO adsorption on the labyrinth surface, only two peaks were identified in TPD; the γ peak was missing (see fig. S4). This agrees with the absence of the dark CO on the labyrinth-like surface, as shown in Fig. 1E.

Several analysis techniques were examined for extracting the adsorption energies from the TPD spectra. The Redhead analysis (*27*) with a prefactor of 10^15^ s^−1^ (an expected value for CO) (*28*) provides a binding energy of 0.43 eV for the bright CO and 0.48 eV for the dark CO. Application of the inversion (*29*) or leading edge (*30*) analyses indicates that the frequency prefactor of the dark CO strongly deviates from the standard value, showing a value of 10^5^ s^−1^ (see fig. S5). Similarly low prefactors have been previously reported on the ferroelectric LiNbO_3_ (*31*–*33*), the possible reasons are discussed below; table ST1 summarizes the results of the various analysis methods.

**Fig. 3.**
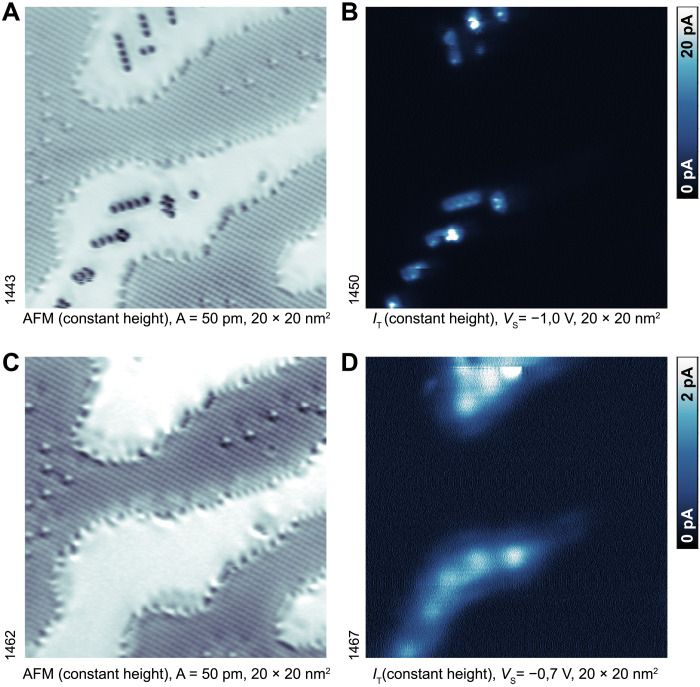
Charge transfer to the dark CO. (**A**) AFM image and (**B**) current image (filled states) of the surface with dark CO. (**C** and **D**) The same region after desorbing the black CO by the STM tip. All images are measured at the same tip-sample distance; frequency and current images were taken concurrently. *I*_T_, tunneling current.

It is unusual to find two chemically different CO adsorption configurations at seemingly equivalent adsorption sites. To understand how this happens, we probed the electronic structure of the adsorbed CO using STM/AFM (Fig. 3). The pronounced state below the Fermi level observed at the dark CO (Fig. 3B) is absent at the bright CO (fig. S2, D to G), suggesting an electronic origin (*34*). Once the CO molecules shown in Fig. 3A were desorbed by the STM tip (scanning at +3.5 V; Fig. 3C), the TaO_2_ terraces exhibit the electronic states characteristic of a clean surface (Fig. 3D) (*20*, *24*), which have been assigned to electrons that originate from the uncompensated surface polarity. We conclude that it is these electrons that bind to the dark CO molecules and that their presence is directly linked to the incipient ferroelectricity and uncompensated surface polarity of the KTaO_3_(001) surface.

**Fig. 4.**
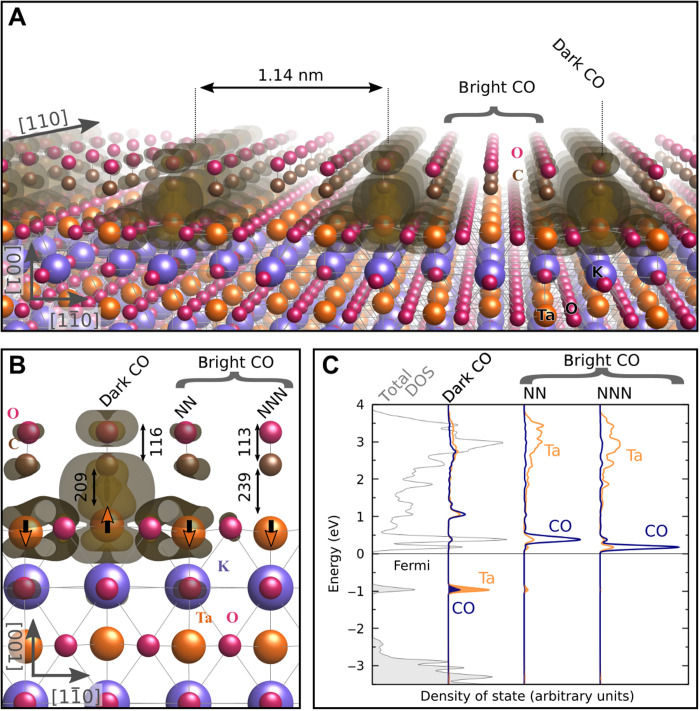
DFT results. (**A**) Calculated structure of the TaO_2_-terminated KTaO_3_(001) surface with adsorbed CO with isosurfaces of the excess charge present within the material’s bandgap. (**B**) Detailed geometry of the adsorbed CO molecules. CO with the excess charge is attributed to the dark CO; the (next) nearest neighbors (N)NN COs are bright. Selected bond lengths are marked (in picometers). Arrows centered on the surface Ta atoms indicate ferroelectric distortions. (**C**) Corresponding density of states (DOS). The leftmost curve (in gray) shows the total DOS, while blue and orange curves show the local DOS for the CO molecule and Ta adsorption site, respectively, as calculated for the dark CO, a nearest neighbor CO, and next nearest neighbor CO (from left to right). Filled (empty) curves represent filled (empty) states.

DFT plus on-site *U* was used to model the TaO_2_ termination of KTaO_3_ with $2\sqrt{2}\times 2\sqrt{2}$ R45° slabs (see Materials and Methods). Several CO adsorption configurations were inspected, including different degrees of interaction with the underlying excess electrons and lattice distortions. TaO_2_-terminated KTaO_3_(001) is a polar surface with an intrinsic excess charge of 0.5 *e* per surface unit cell. If this charge forms a 2D electron gas, then it is associated with polarization of the near-surface layers: The surface Ta atoms of a clean TaO_2_-terminated surface are buckled down by 21 pm with respect to the surface O atoms due to the uncompensated surface polarity (*20*). The lowest-energy configuration for a full monolayer (ML) of CO is shown in Fig. 4A: The excess charge from the uncompensated polarity localizes on a row of CO molecules arranged along <110> directions, forming bipolarons coupled with the CO molecules. Bader analysis shows a strong hybridization: 0.4 *e* is transferred to antibonding orbitals of the CO molecule. This configuration explains the experimentally observed dark CO and is associated with a locally reversed buckling of the underlying Ta atom, by +11 pm with respect to the plane of the surface O atoms (Fig. 4B). We note that the vertical positions of the bright and dark CO molecules remain almost identical, because the effect of the Ta buckling is compensated by the length of the Ta─C bond.

The remaining CO molecules (bright) in the slab do not carry considerable excess charge and are chemically similar to CO in the gas phase. The calculated C─O bond length for the bright CO is 113 pm (gas-phase CO has 112.8 pm) (see Fig. 4B) (*35*, *36*). The dark CO has a calculated bond length of 116 pm, which indicates a substantial weakening of the C─O bond, i.e., activation of the CO. The effect originates from electron donation into the CO π* antibonding orbital (see the density of states in Fig. 4C), and its magnitude appears comparable to a CO molecule adsorbed on catalytically active metals, such as Pt or Pd (*35*, *37*). Accordingly, the dark CO is located closer to the Ta atom than the bright CO, at 209 and 239 pm, respectively. A similar coupling of CO molecules with (single-electron) polarons has been previously reported on TiO_2_(110) surfaces (*38*, *39*), but the effects observed on KTaO_3_ are an order of magnitude stronger due to the stronger charge localization of bipolarons with respect to polarons and to the coupling with ferroelectric distortions. A systematic analysis of various options of coupling the CO molecule on the TaO_2_ termination with single-electron polarons and dispersed charge (fig. S6) returned less stable configurations. The bipolaronic configuration is also consistent with the experimentally observed attractive coupling of dark CO molecules along the <110> directions through the associated ferroelectric buckling in the substrate. The coupling of the CO molecules with the bipolarons and the consequent local reversal of the Ta buckling leads to a switching of the electrostatic potential above these molecules (fig. S8), consistent with the dark (attractive) AFM signal.

The bipolaron scenario is also consistent with the number of electrons expected in the substrate. The experimentally observed arrangement of the dark CO is approximately 3√2 × √2 R45°, which corresponds to a charge density of 1/3 *e* per unit cell. This is comparable to 1/2 *e* per surface unit cell predicted by the polar catastrophe scenario (assuming infinitely large terraces).

While all the aforementioned arguments support the bipolaronic configuration of the dark CO, a significant inconsistency arises in the binding energies. The calculated adsorption energy of bright CO, *E*_bright_ = −0.50 eV, agrees with the desorption barrier of 0.43 eV derived from the TPD analysis. However, desorbing the dark CO costs *E*_dark_ = −1.32 eV according to DFT. The computed adsorption energy is rigid with respect to variations of the computation parameters within any reasonable limits and markedly exceeds the value of 0.48 eV derived from TPD.

## DISCUSSION

There are indications of unconventional processes involved in desorption of the dark CO, such as the frequency prefactor of 10^5^ s^−1^ derived from advanced analysis of TPD data (fig. S5). In transition state theory (*40*), the low prefactor suggests a very low entropy of the transition state, indicating that the state of the substrate required to bind the dark CO is extremely unlikely at the desorption temperature. STS measurements on a clean surface show that the density of states within the bandgap of the TaO_2_ termination strongly decreases above *T* = 80 K (see fig. S7). This indicates that the excess electrons required by the dark CO become unavailable above a certain temperature threshold, and the dark CO converts into the weakly bound bright CO. This could be due to a phase transition where the polarity compensation mechanism changes to a different scheme. Then, close to the desorption temperature, the excess charge and the outward buckling of Ta are stabilized only by the presence of the dark CO, which also explains why the DFT value of the adsorption energy (calculated for 0 K) does not apply for desorption.

The observed behavior of the bright and dark CO has direct implications for catalysis. It is widely assumed that the degree of molecule chemical activation is proportional to its binding strength toward the substrate (Sabatier principle) (*19*, *41*), and it was theoretically predicted that ferroelectric materials can overcome this limitation (*11*, *42*). On the KTaO_3_(001) surface, two CO adsorption configurations coexist that desorb at comparable temperatures but have different chemical properties directly detectable by AFM. This duality is enabled by the possibility to displace the Ta atoms from their equilibrium positions and by the associated changes in the orbital occupancy and hybridization. The concept therefore relies on the unique properties of (incipient) ferroelectrics (*43*, *44*). The model case of CO adsorption also sheds light on possible fundamental processes occurring at polar surfaces, which play a key role in pyroelectric and flexoelectric catalysis where rapid polarization switching manipulates adsorption states.

## MATERIALS AND METHODS

The AFM/STM experiments were performed at *T* = 4.8 K in an ultrahigh vacuum (UHV) chamber with a base pressure below 2 × 10^−9^ Pa, equipped with a commercial Omicron q-Plus LT head and a custom-design cryogenic preamplifier (*45*). Tuning fork sensors with a separate wire for the tunneling current were used, with a resonance frequency *f*_0_ ≈ 32 kHz and quality factor *Q* ≈ 20,000. Etched tungsten tips were cleaned by self-sputtering in argon gas (*46*) and treated on a Cu(110) surface to ensure their metallic character. The tip was subsequently functionalized by a CO molecule on the CO-exposed KTaO_3_(001) surface. Short-range force-distance forces were calculated using the Sader formula (*47*) after subtracting the long-range component (*48*). Controlled annealing of the sample was done in a manipulator, applying 10 min for equilibrating the temperatures. The quoted temperatures are accurate within ±10 K.

The TPD measurements were carried out in a separate UHV system (*49*) with a base pressure of 5 × 10^−9^ Pa. The sample was mounted on a Ta back plate, cooled by a Janis ST-400 UHV liquid-He flow cryostat, and heated by direct current through the back plate. The temperature was measured by a K-type thermocouple spot-welded to the sample plate; the temperatures are accurate within ±10 K. Isotopically labeled ^13^CO was dosed by an effusive molecular beam with a hat-shape profile (*49*, *50*). The spot position was calibrated by dosing a large amount of water before the in situ cleaving. This resulted in a visible, circular patch of water ice on the sample. Its diameter was ~3.5 mm on a sample of ~4 × 5 mm^2^ in size. The combination of isotopically labeled ^13^CO and a precisely dosed beam spot suppressed any contribution from background CO. A linear temperature ramp of 0.2 K/s was used. Such a low ramp speed is necessary to minimize the temperature gradient inside the sample and allows measuring the temperature by a thermocouple spot-welded to the sample holder. A HIDEN quadrupole mass spectrometer in a line-of-sight configuration was used for detection of the TPD flux. One ML is defined as one molecule per surface unit cell, i.e., 6.3 × 10^14^ cm^−2^. We assumed first-order Arrhenius kinetics and a coverage-dependent desorption activation energy.

Synthetic single-crystalline KTaO_3_ samples were prepared by solidification from a nonstoichiometric melt. Trace impurities of Ba, Cu, and Yb in atomic concentrations of ~10^−4^ were used to ensure n-type electrical conductivity. After introducing to the vacuum chamber, the samples were outgassed at 600 to 800 K and cleaved at *T* = 265 K, using a tungsten-carbide blade (*20*).

The DFT calculations were performed by using the Vienna Ab initio Simulation Package (VASP) (*51*, *52*). We adopted the strongly constrained and appropriately normed meta-generalized gradient approximation (SCAN) (*53*) with the inclusion of van der Waals interactions (*54*) and an on-site effective *U* (*55*) of 4.0 eV on the d orbitals of Ta atoms (in agreement with constrained random phase approximation calculations on the bulk material) (*24*). The TaO_2_ termination of KTaO_3_(001) was modeled by using 2√2 × 2√2 slabs, with six TaO_2_ layers alternated to five KO layers, in a symmetric setup [mirror symmetry on the central (001) KO layer], including a vacuum region of more than 3 nm (see also fig. S9). All the atomic sites (except those on the central KO layer) were relaxed using standard convergence criteria (residual forces smaller than 0.01 eV/Å), with a plane-wave energy cutoff of 500 eV and a 3 × 3 × 1 *k*-point grid.

The CO adsorption was modeled (symmetrically on both sides of the slab) considering (i) one ML coverage with 25% bipolaronic dark CO aligned along <110> and 75% bright CO molecules and (ii) 25% coverage including exclusively bipolaronic dark CO molecules aligned along <110>. The adsorption energy was calculated as energy difference between the systems in (i) and (ii) for the bright CO, while for dark CO, it was calculated as energy difference between (ii) and the clean surface (referenced to the energy of CO molecules in the gas phase). The reference clean surface hosts a mixed configuration of bipolarons and polarons that represents the ground state arrangement of localized charge on the clean surface (see fig. S6). Adsorption in different solutions for the in-gap states (single-electron polarons and dispersed electrons) was also considered, as shown in the Supplementary Materials. All solutions were obtained by adopting different initial conditions for local structure, electronic density, and wave function in unconstrained self-consistent calculations (*24*). We used VESTA (*56*) to show the spatial distribution of the charge density of the in-gap states.

## Supplementary Materials

### This PDF file includes:

Supplementary Text Figs. S1 to S9 Tables ST1 and ST2
