# Supplementary material for: Surface chemistry on a polarizable surface: Coupling of CO with KTaO3(001)
Source: Sci Adv. 2022 Aug 19;8(33):eabq1433. doi: 10.1126/sciadv.abq1433 (PMC9390988; doi:10.1126/sciadv.abq1433)
Supplement: Supplementary file 1 — Supplementary Text Figs. S1 to S9 Tables ST1 and ST2 [file sciadv.abq1433_sm.pdf]

Science Advances
AAAS

Supplementary Materials for

Surface chemistry on a polarizable surface: Coupling of CO with $KTaO_3(001)$

Zhichang Wang *et al.*

Corresponding author: Martin Setvin, martin.setvin@mff.cuni.cz; Cesare Franchini, cesare.franchini@unnivie.ac.at

Sci. Adv. **8**, eabq1433 (2022)
DOI: 10.1126/sciadv.abq1433

This PDF file includes:

Supplementary Text
Figs. S1 to S9
Tables ST1 and ST2

Supplementary Text

CO adsorption at the KO termination

CO adsorption at KO terraces is weak with the corresponding desorption peak at T≈75 K (Fig. 2A). We note that this peak is at the onset of the TPD spectra, thus the exact peak shape is likely affected by effects associated with initiating the temperature ramp.

The adsorption geometry of CO on the KO termination is incommensurate with the substrate (Fig. S1) and the apparent arrangement of CO molecules is close to a hexagonal symmetry with a nearest neighbor distance of approximately 0.28 nm.

Images of CO on the TaO_2 termination

Fig. S2a-c illustrates the distance dependence of AFM images of the bright and dark CO. The same region is shown in all three cases, with small lateral shifts. Fig. S2d,e shows the influence of the tunneling current on the stability of the system. When a small sample bias V_s is applied, tunneling current flows only through the dark CO and induces apparent hopping of the molecules (horizontal streaks, apparently adjacent dark CO molecules. This apparent hopping occurs only when both the bright and dark CO molecules are present simultaneously; the system is stable when solely dark CO is present, such as in Fig. 4 of the main text. This indicates the apparent motion in Fig. S2d is based on switching between the neighboring bright and dark configurations.

Fig. S3 shows partial ordering of the dark CO molecules along the <110> directions. We note that the bright spots present on the KO terraces in most images shown in this work have been previously assigned as oxygen vacancies (marked V_O)[20] and they have no relation to CO adsorption discussed here.

TPD measurements on the “labyrinth” surface

In order to illustrate the coupling between the dark CO and excess electrons, we have performed a series of experiments on the so called “labyrinth” termination, which is prepared by UHV annealing the as-cleaved surface to temperatures above 150°C. This termination does not possess electronic states in the band gap because the terraces are too narrow; so the opposite excess charges of the KO and TaO_2 terminations cancel out and the variation of electrostatic potential is not strong enough to induce the polar catastrophe.

TPD data from this termination (see Fig. S4a) show only two peaks, the lower peak (α) at 75 K corresponds to desorption from the KO termination and the other peak (β) centered at 130 K originates from desorption of the bright CO from TaO_2 terraces. The shoulder related to dark CO is absent (γ in the main text).

The TPD data are supplemented by AFM images of this termination after dosing a saturation amount of CO at $T = 100$ K (Fig. S4b), and after annealing to $T = 138$ K (Fig. S4c), which confirms the identification of the TPD peaks (see also Fig. 1D, E of the main text).

An interesting aspect in the TPD curves in Fig. S4a (also in Fig. 2A in the main text) is that both peaks grow simultaneously with the increasing coverage. This is in contrast with normal behavior where high-temperature peaks are filled first. We have repeated these experiments and excluded experimental artefacts. We attribute this behavior to existence of an energy barrier for diffusion of the CO molecules across the steps between the two terminations.

Analysis of TPD data

To find the value of the prefactor, v, that gives the best agreement between the experimental results and simulation we calculated the error χ^2 for each peak defined as a square of the difference between experimental and simulated curve summed for all curves belonging to the given peak. The results in Figs. S5c, e show the dependence of χ^2 as a function of v, indicating the optimum values of the prefactor. We get similar results if we choose another lower-coverage curve instead of the curve used in Figs. S5a, c for simulation. The temperature range for the analysis is limited as displayed in Figs. S5a, c. Edges of individual peaks are 'cut off' directly without subtraction, so as not to include these data in the error analysis and maximize the contribution from the analyzed peak.

The TPD data have been analyzed by several techniques, results are summarized in Table ST1. Methods such as the inversion analysis or leading-edge analysis consistently show frequency prefactors that significantly deviate from the standard values expected for small molecules.[28] Similarly low prefactors in order of 10^6 have been previously observed for adsorption on the ferroelectric $LiNbO_3$.[30,31] This has been interpreted as a temperature-dependent binding energy in the vicinity of the desorption peak, assuming a linear scaling. Our data are consistent with such scenario. The dark CO depends on the existence of excess electrons in the substrate. Our observations (Fig. S7) indicate that these electrons become unavailable at temperatures comparable to the desorption peak, which would explain a temperature-dependent binding.

In such a case, desorption cannot be explained by a standard first-order process, where each molecule desorbs independently due to the activation energy supplied to the molecule. Desorption occurs due to changes in the substrate (like a phase transition). Therefore, standard TPD analyses do not yield the actual desorption energy. Experimentally we can only estimate a lower limit for the magnitude of the desorption energy of the dark CO: Its adsorption must be stronger than that of the bright CO, since the dark CO remains adsorbed at temperatures where the bright CO has been desorbed.

Systematic DFT analysis of the CO configurations

We considered different scenarios for the adsorption of CO molecules on the TaO_2 terrace. Figure S6 shows the most relevant cases, including different arrangements of the excess electronic charge on the surface (Fig. S6a-e).

The ground-state polaronic configuration of the clean surface is shown in Fig. S6f. This configuration was used as a reference to calculate the adsorption energies E, as described in the Methods Section.

The most stable adsorption configuration in the $2\sqrt{2} \times 2\sqrt{2}$ computational cell is given by <110> oriented rows of bipolarons hybridizing with the CO molecule on top of the Ta atom, associated with a local reversal of the ferroelectric-like polarization: This scenario can be obtained

for both high (*e.g.,* 1 ML, see main text) and low (Fig. S6a) CO coverage, *i.e.*, with and without the bright CO. These two cases were used to calculate the adsorption energy of the bright CO, by comparing the total energies of the systems (see Methods Section).

Other arrangements of bipolarons lead to less favorable adsorption energy: by arranging the bipolarons along the <100> directions, as shown in Fig. S6b, the stability is reduced by 130 meV per (dark) CO molecule as compared to the <110>-oriented rows.

We considered also the coupling of single-electron $5d^1$ polarons (rather than $5d^2$ bipolarons) with CO molecules. Figs. S6c,d show the results for high and low CO coverage, respectively. The hybridization with the CO molecule occurs with one *d* orbital of Ta (either d_{xz} or d_{yz}), at variance with bipolarons where both the bipolaoronic d_{xz} and d_{yz} orbitals hybridize with the adsorbate. As mentioned in the main text, this scenario leads to a less favorable adsorption energy by ≈150 meV for the dark CO, and to a different arrangement of the dark CO molecules as compared to the experimental AFM measurements.

Finally, we considered the adsorption in the absence of any localized state. Fig. S6e shows 1 ML of CO molecules adsorbing on the surface hosting a two-dimensional electron gas (2DEG). In this case, all molecules are equivalent and the adsorption energy is even weaker (−0.6 eV); the best configuration for the same excess charge in Fig. 4 of the main text corresponds to an average adsorption energy of −0.7 eV.

Stability of the in-gap states at elevated temperatures

The adsorption energy of the dark CO calculated by DFT is apparently inconsistent with the TPD measurements (–1.32 vs. –0.48 eV). This can be explained by the excess electrons required for the stabilization of the dark CO configuration becoming unavailable above a certain temperature threshold. This is supported by temperature-dependent measurements of the tunneling current above the clean surface shown in Fig. S7. All data were measured with a Cu-terminated tip prepared by scanning a Cu(110) surface. The tip-sample distances were comparable for all experiments, which is estimated from the atomic contrast in AFM images.

While the magnitude of the tunneling current is highest at LHe temperatures, it dramatically drops above the LN_2 temperature, and almost vanishes at T=109 K (last row of Fig. S7). This observation has been made many times at multiple samples. The tunneling current at −0.5 V sample bias is related to in-gap states, which originate from the uncompensated polarity of the $KTaO_3$(001) surface and it is possible that the polarity-compensation mechanism undergoes a change at temperatures above ~80 K. This can explain the limited stability of the dark CO observed by TPD, in spite of very strong adsorption at low temperatures.

Origin of the dark-bright contrast inversion in the AFM measurements.

Our DFT calculations can help to identify the origin of the different (dark and bright) signals measured by the AFM. We exclude this effect to be topographic in origin, since in the calculations all CO ad-molecules show terminating O atoms at essentially the same height (independently of the coupling with the underlying bipolaron and Ta buckling, as shown in Fig. 4B in the main text). The inversion of the AFM contrast seems mostly due to the local electrostatic potential. Figure S8 shows a local inversion of the electrostatic potential above the Ta+CO bipolaron (the dark CO). This effect arises from the negative bipolaronic charge, which is partially

located on the CO molecule and modifies the molecular dipole moment. Consistently, all other CO admolecules show no inversion of the local electrostatic potential (see Fig. S8B).

In order to further investigate the origin of the inversion of the electrostatic potential, we performed additional calculations to decouple the impact of the bipolaronic charge transfer from the local reversal of the Ta buckling. By starting from a fully relaxed slab with dark CO molecules, we moved progressively inwards the underlying Ta atom, while letting the surrounding atoms relax (all other ions constrained to the initial positions). With this approach we managed to obtain (at the cost of 1.1 eV) a Ta buckling of -17 pm with respect to coordinated surface O atoms, similar to buckling of Ta atoms on the clean surface (-22 pm). Bader charge analysis shows that the charge transfer of the bipolaron to the CO is practically unaffected (as compared to a standard dark CO), as are the structural properties of the molecule. In this setup, the switching of the electrostatic potential still occurs, however, the peak is damped from approximately +0.8 eV to +0.5 eV. This result indicates that the switching of the electrostatic potential cannot be explained exclusively in terms of the local reversal of the Ta buckling. Therefore, the contribution of the bipolaronic charge transfer seems to be a key contribution to the switching.

Setup of the DFT slabs.

Figure S9 shows an example of the slabs used in the DFT calculations. As described in the main text, we adopted symmetric slabs, with a mirror plane (parallel to the surface terminations) on the central atomic layer. The CO adsorption was modeled by maintaining this symmetry. Additional setups were also investigated (e.g., by asymmetrically adsorbing the CO molecules only on one side of the slab) with no sizable impact on the results and analysis presented in the main text. Similarly, adsorption energies calculated using different lateral sizes of the unit cells (i.e., $4\sqrt{2}\times2\sqrt{2}$ and $\sqrt{2}\times4\sqrt{2}$) show no significant difference (as expected, considering that the density of excess charge on the surface is constant, i.e., 0.5e per 1×1 unit cell). These slabs also confirmed the arrangement of bipolarons along <110> directions as the most stable bipolaronic pattern.

The large number of atomic layers in our slabs was chosen to reduce to a negligible extent the interaction between the electronic states on the two different termination sides of the slab, as reported in our previous study (Ref. 24). This setup also guarantees sufficient space for the decay of the ferroelectric-like distortions in the surface and subsurface layer of the TaO_2 terminations towards deeper layers, which remain undistorted (as expected for the bulk $KTaO_3$ that is known to be incipient-ferroelectric).

Fig. S1.

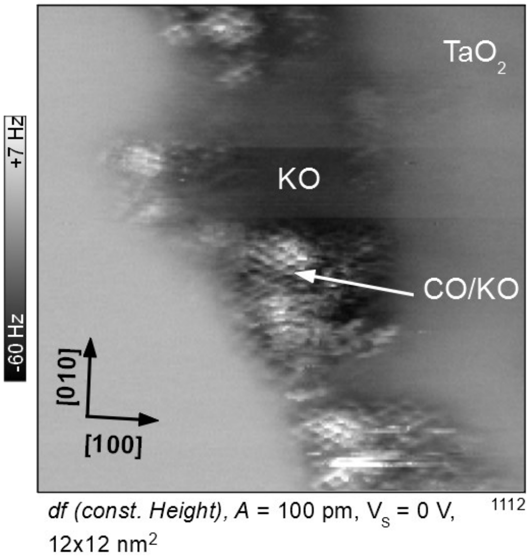

Figure S1: CO on KO termination. AFM image of the CO molecules adsorbed at the KO termination. Measured at $T = 51\,K$.

Fig. S2.

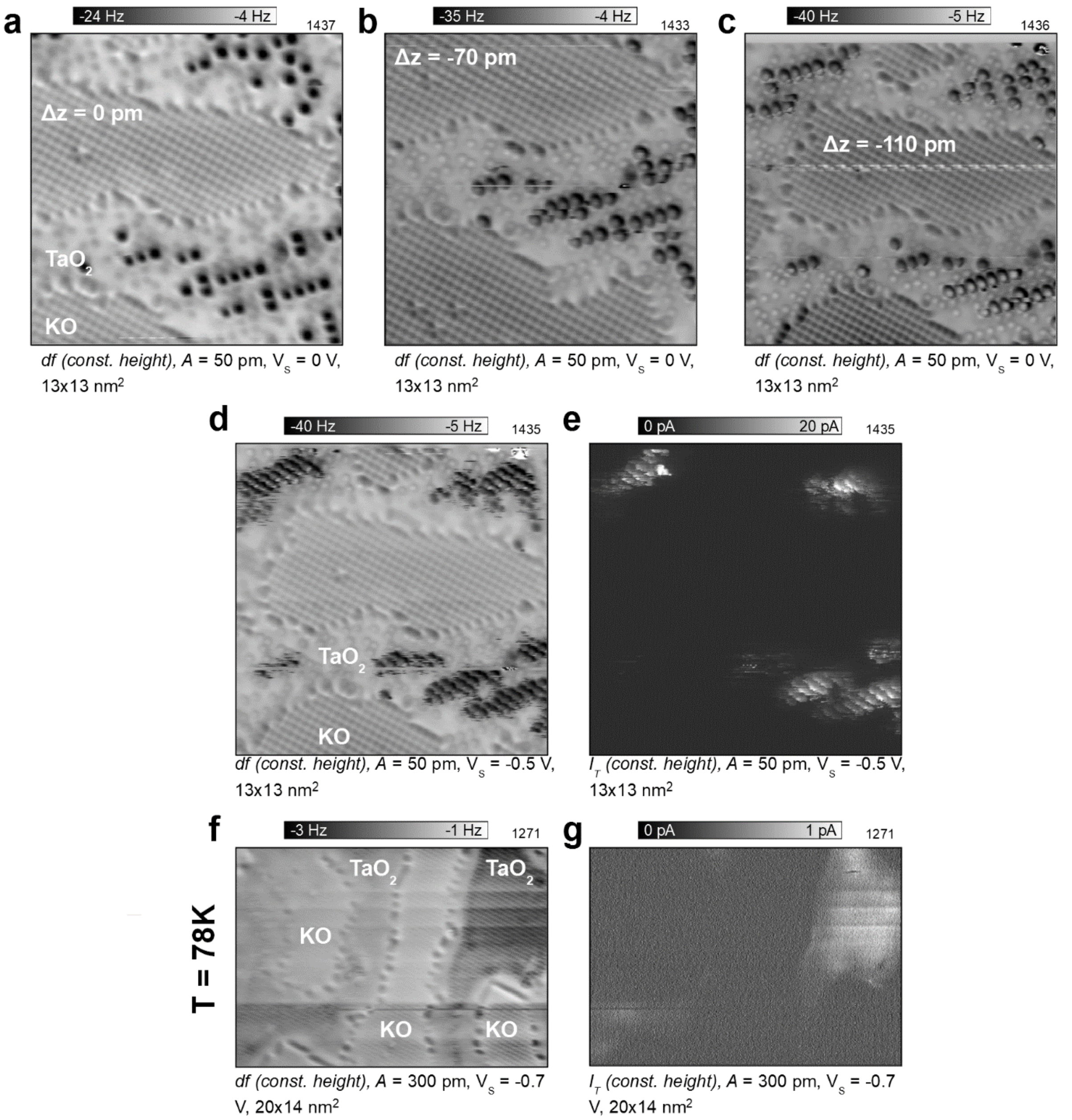

Figure S2: Imaging the dark CO at various conditions. a,b,c) An area containing bright and dark CO molecules imaged at different tip-sample distances (marked in the image). Measured at zero bias and T=4.8 K. d,e) The same area when a negative sample bias of -0.5 V is applied. The tunneling current is detected only above the dark CO molecules and the tunneling induces apparent migration of the dark CO. f,g) Saturation coverage of CO adsorbed and measured at T=78 K. The dark CO appears mobile at this temperature but it stays confined in central regions of larger TaO_2 terraces (where excess electrons are available due to the surface polarity[20]).

Fig. S3.

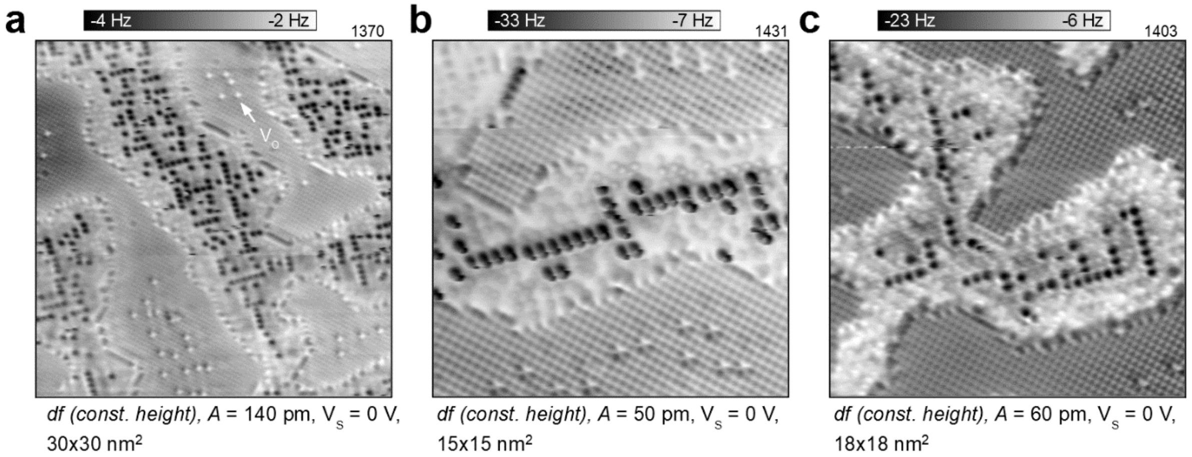

Figure S3: Spatial distribution of the dark CO. A preference for arrangement along the <110> directions is observed systematically in different experiments.

Fig. S4.

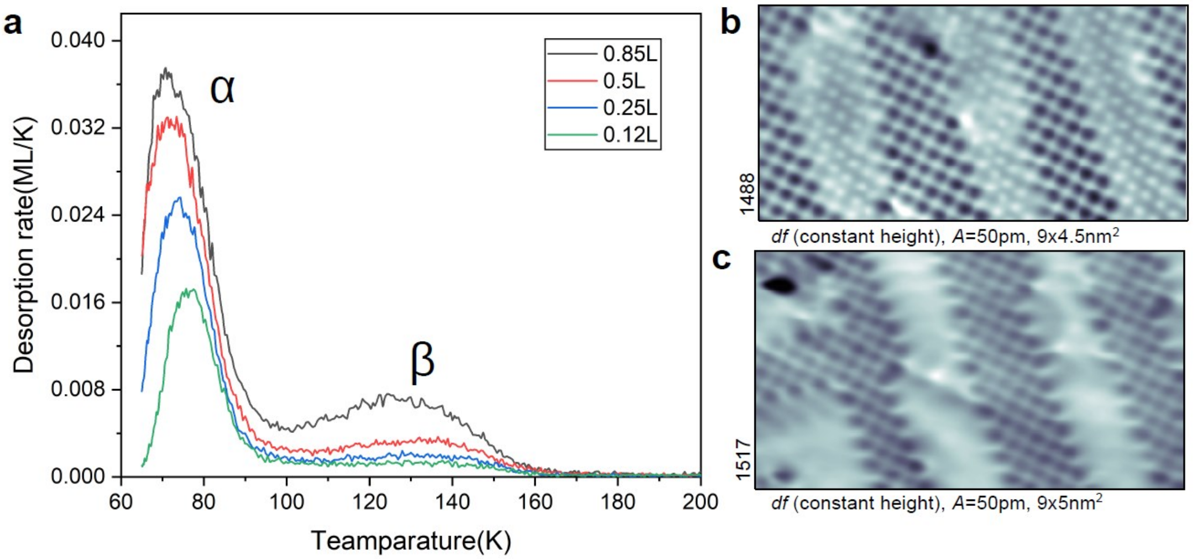

Figure S4: TPD data from the labyrinth structure.[20] a) Coverage-dependent desorption curves. Desorption peaks from the KO and TaO_2 terminations are marked α and β, respectively. b) AFM image of the labyrinth surface with the TaO_2 terraces saturated by CO. c) The labyrinth surface after desorbing the CO by annealing to 138 K for 10 minutes.

Fig. S5.

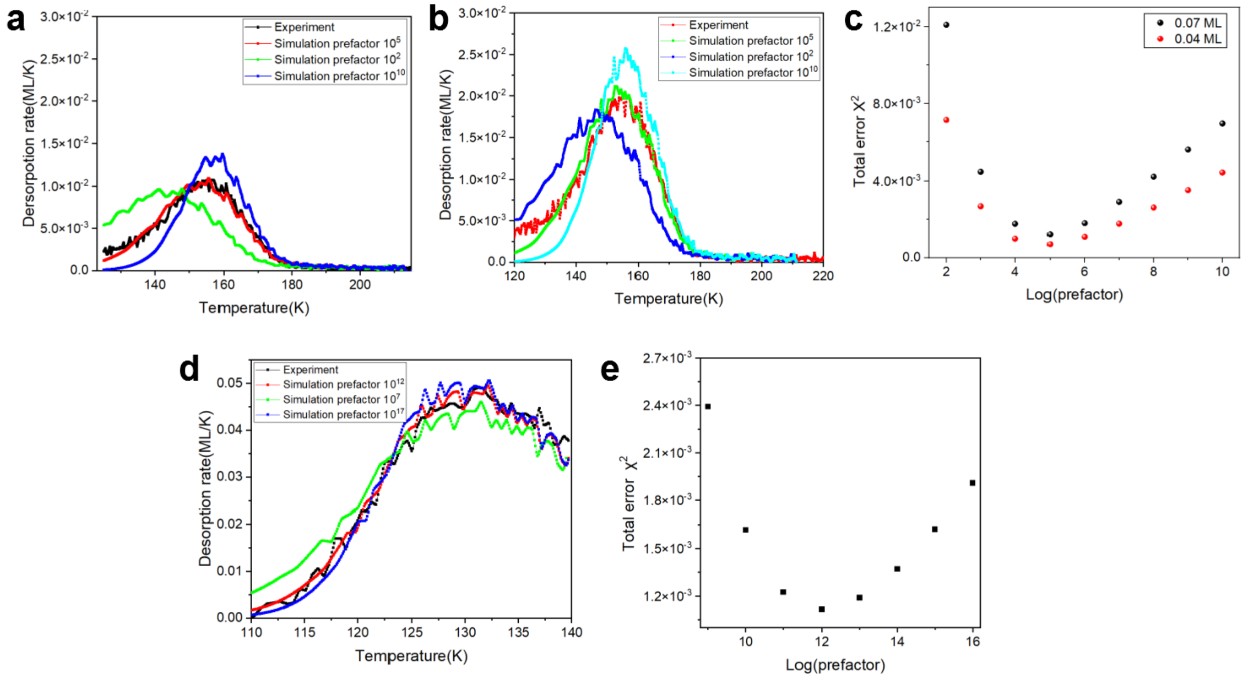

Figure S5: Inversion analysis of the TPD data in Fig. 2A of the main text. (a, b) Desorption curves of the dark CO, showing experimental data from 0.04 and 0.07 ML of adsorbed CO. Fits are shown for the optimum value of the frequency prefactor ($v_0 = 10^5$ s^{-1}), as well as for prefactors several orders of magnitude lower and higher. c) The total deviation between the experimental and simulated data, plotted as a function of the frequency prefactor. (d,e) The same procedure for the bright CO, showing an optimal match for $v_0 = 10^{12}$ s^{-1}.

Fig. S6.

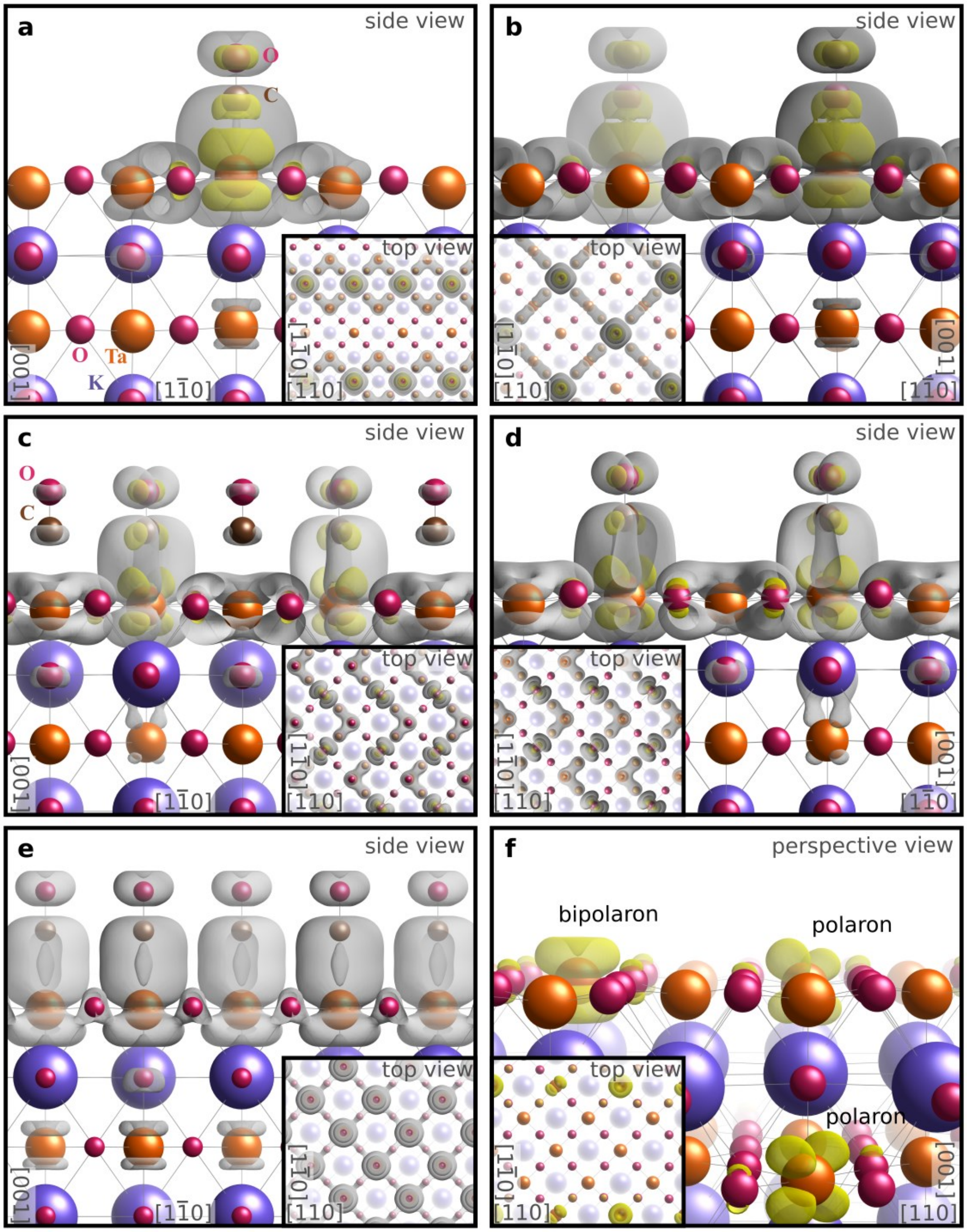

Figure S6: DFT analysis of the CO configurations. Side views (and top views in the insets) for bipolarons on Ta+CO arranged along <110> (panel a) and along <100> (panel b), for single-electron polarons on Ta+CO at high (panel c) and low (panel d) coverage, and for CO molecules adsorbing on the 2DEG (panel e). The polaronic configuration used as reference for calculating the adsorption energies *E* is shown in panel f. The isosurfaces represent the charge density of the excess charge on the TaO_2 surface (grey and yellow colors indicate low and high levels, respectively).

Fig. S7.

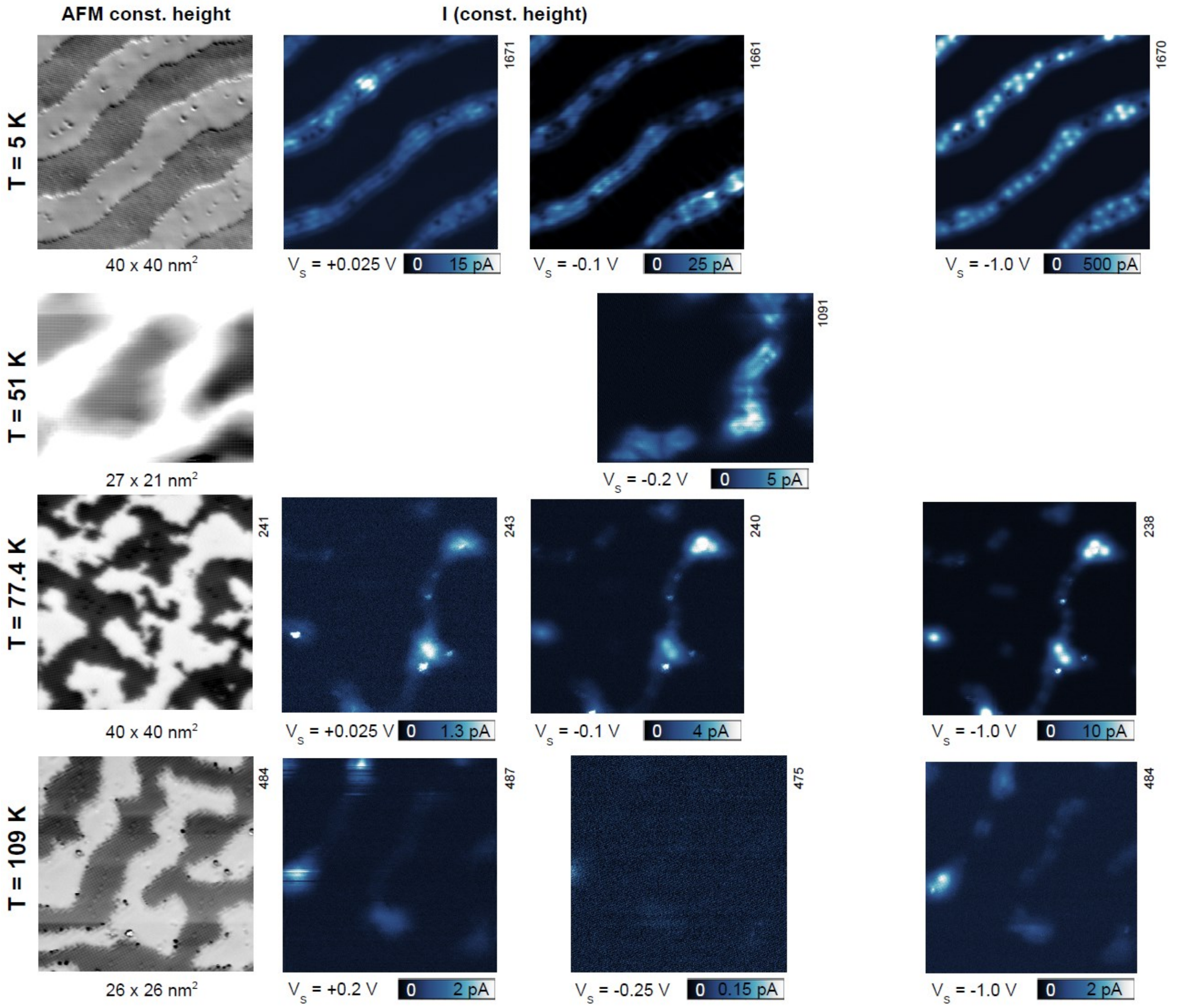

Figure S7: Electronic structure of the clean cleaved $KTaO_3$ (001) surface at various temperatures. The temperature increases from top to bottom. The left row shows an AFM image (constant height), demonstrating the surface topography. Images in the right part show the tunneling current recorded at several representative sample voltages. The magnitude of the tunneling current decreases with increasing temperature.

Fig S8

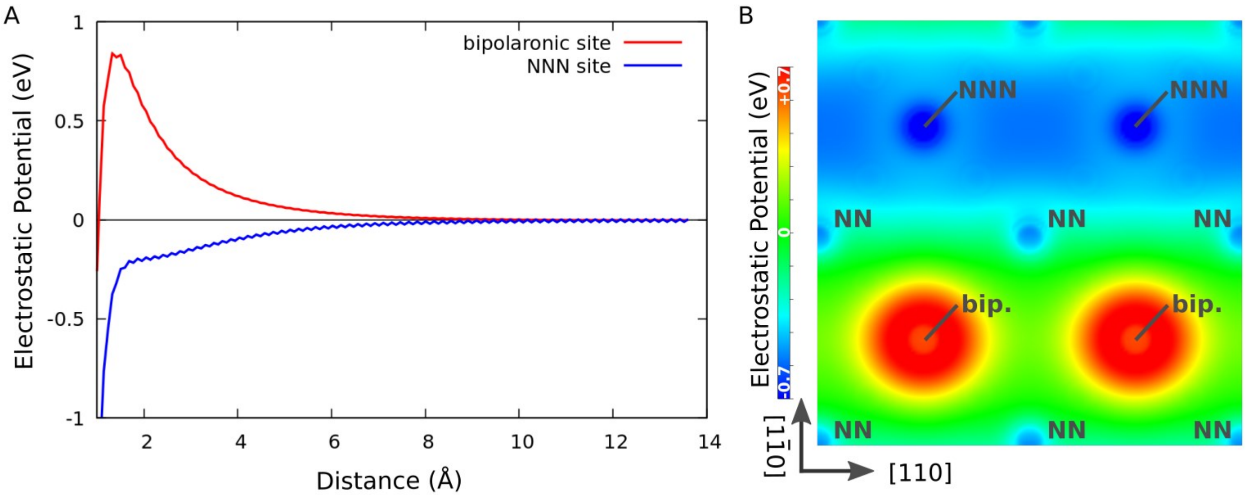

Fig. S8: Electrostatic potential for electrons obtained by DFT calculations on the full CO monolayer. The line profile in panel A is taken above a CO molecule coupled with a bipolaronic Ta atom (red line), and above a CO molecule furthest away (next nearest neighbor, NNN) from the bipolaronic site (blue line). The distance axis is referred to the position of the terminating O atom of the admolecule. Panel B shows a map of the electron electrostatic potential on a plane parallel to the surface, approximately 1.1 Å above the terminating O atoms of the CO admolecules. The position of the bipolaronic, nearest neighbor and next nearest neighbor sites are indicated with bip., NN and NNN, respectively.

Fig S9

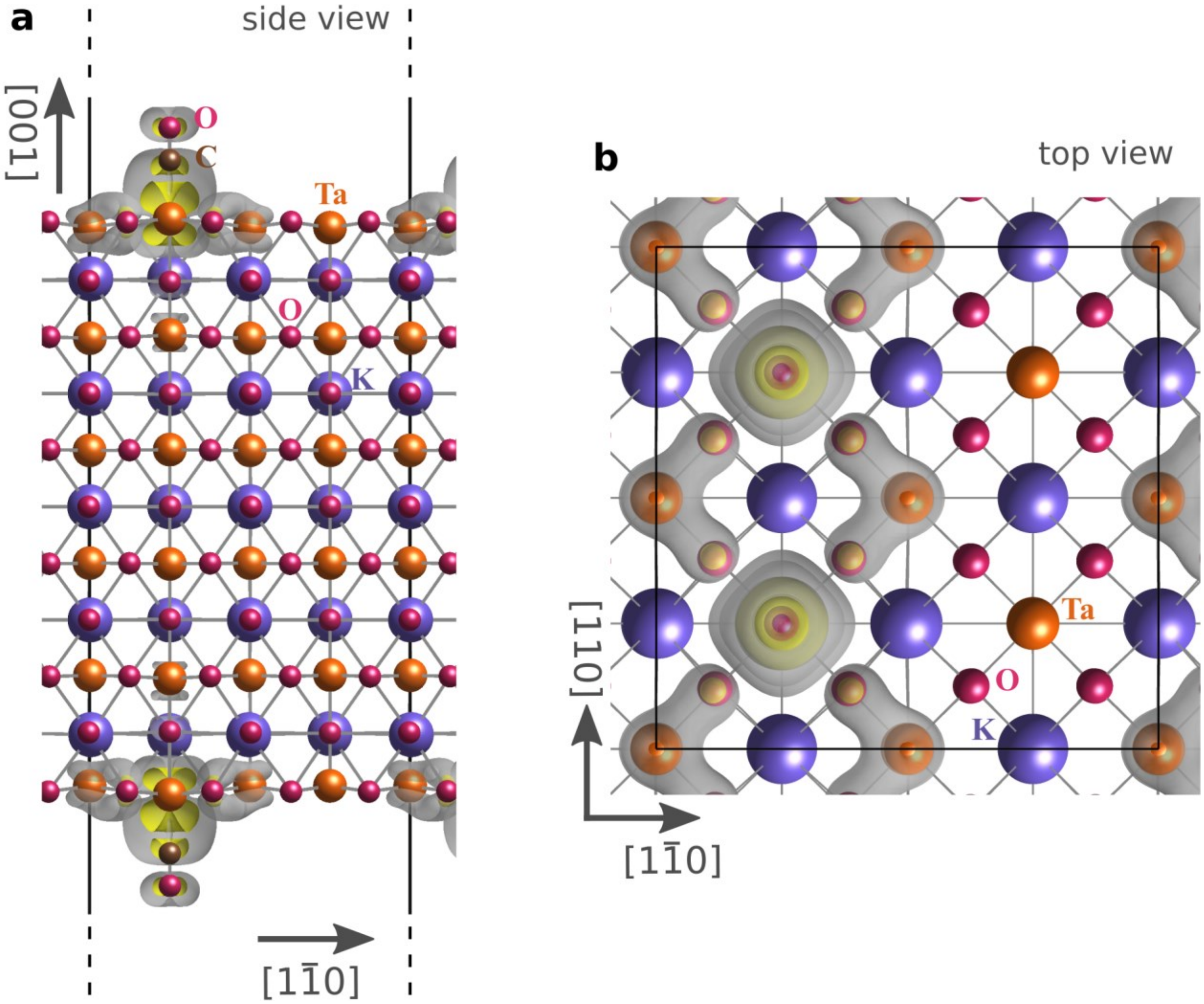

Fig. S9: Example of a slab used in the DFT calculations. The side and top views shown in panel a and b, respectively, represent the slab used in the calculations of low CO coverage, with all CO coupling with bipolarons (black lines indicate the unit cell). Both sides of the symmetric slab are shown (a part of the vacuum region is cut).

Table ST1

	'bright' CO		'dark' CO	
	ν_0 (s^{-1})	E_{ads} (eV)	ν_0 (s^{-1})	E_{ads} (eV)
Redhead	10^{15}	−0.43	10^{13}	−0.48
Inversion analysis	10^{12}	−0.35±0.05	10^{5}	−0.23±0.03
Leading edge	---	---	10^{0}	−0.08±0.04
DFT		−0.50		−1.32 (bipolaron)<br>−0.88 (polaron)

Table ST1: Analysis of the experimental data by different techniques and compared to calculated adsorption energies.

Table ST2.

	E_{bright} (eV)	E_{dark} (eV)
CO + bipolaron (Figs. 4 and S6a)	−0.497	−1.32
CO + polaron (Figs. S6c and S6d)	−0.476	−0.878
2DEG (Fig. S6e)	−0.60	

Table ST2: Computed adsorption energies for different coupling of the CO to polarons. The reference energy for the dark CO is the ground state shown in Fig. S6f.
